# Hepatitis C Screening and Antibody Prevalence Among Newly Arrived Refugees to the United States, 2010–2017

**DOI:** 10.1007/s10903-023-01471-8

**Published:** 2023-03-30

**Authors:** Kailey Urban, Colleen Payton, Blain Mamo, Hannah Volkman, Katherine Giorgio, Lori Kennedy, Yuli Chen Bomber, Kristine Knuti Rodrigues, Janine Young, Carol Tumaylle, Jasmine Matheson, Azadeh Tasslimi, Jessica Montour, Emily Jentes

**Affiliations:** 1 Minnesota Department of Health, Infectious Disease Epidemiology, Prevention, and Control Division, St. Paul, MN 55164 Türkiye; 2 School of Nursing and Public Health, Moravian University, Bethlehem, PA U.S.; 3 Department of Family and Community Medicine, Thomas Jefferson University, Philadelphia, PA USA; 4 Colorado Department of Public Health and Environment, Newcomer Health Program, Health Equity Branch, Disease Control and Public Health Response Division, Denver, CO USA; 5 Denver Health and Hospitals, Department of General Pediatrics, University of Colorado School of Medicine, Denver, CO USA; 6 Colorado Department of Human Services, Colorado Refugee Services Program, Denver, CO USA; 7 Washington State Department of Health, Office of Communicable Disease Epidemiology, Refugee and Immigrant Health Program, Shoreline, WA USA; 8 U.S. Committee for Refugees and Immigrants, Refugee Health Services, Austin, TX USA; 9 National Center for Emerging and Zoonotic Infectious Diseases, Division of Global Migration and Quarantine, Centers for Disease Control and Prevention, Atlanta, GA USA

**Keywords:** Hepatitis C, Screening, Refugee Health, Immigrants

## Abstract

Six refugee screening sites collaborated to estimate the prevalence of hepatitis C virus (HCV) antibodies among newly arrived refugees in the United States from 2010 to 2017, identify demographic characteristics associated with HCV antibody positivity, and estimate missed HCV antibody-positive adults among unscreened refugees. We utilized a cross-sectional study to examine HCV prevalence among refugees (N = 144,752). A predictive logistic regression model was constructed to determine the effectiveness of current screening practices at identifying cases. The prevalence of HCV antibodies among the 64,703 refugees screened was 1.6%. Refugees from Burundi (5.4%), Moldova (3.8%), Democratic Republic of Congo (3.2%), Burma (2.8%), and Ukraine (2.0%) had the highest positivity among refugee arrivals. An estimated 498 (0.7%) cases of HCV antibody positivity were missed among 67,787 unscreened adults. The domestic medical examination represents an opportunity to screen all adult refugees for HCV to ensure timely diagnosis and treatment.

## Introduction

### Hepatitis C Epidemiologic Overview

Hepatitis C virus (HCV) infects the liver and can lead to cirrhosis, hepatocellular carcinoma (HCC), the need for liver transplantation, and death. Global prevalence estimates of chronic hepatitis C infection during 2015 were approximately 1%, with variation between and within countries [1]. The prevalence of hepatitis C antibodies in the adult United States population during 2013–2016 was estimated at 1.7% (95% CI: 1.4 − 2.0%) [2]. HCV infection is diagnosed via testing for anti-HCV antibodies followed by a nucleic acid test for HCV ribonucleic acid (RNA) to confirm chronic infection in those who tested positive for anti-HCV antibodies. It is important to test for hepatitis C infection as the majority of infected people develop chronic viremia, most people do not experience symptoms or symptoms are nonspecific, and 90% of people can be cured with treatment in 8 to 12 weeks [3].

### Refugee Resettlement in the United States and the Domestic Medical Examination

There were 600,898 refugees who arrived in the United States between 2010 and 2020 [4]. The Centers for Disease Control and Prevention (CDC) recommends that all newly arrived refugees receive a domestic medical examination (DME) within 30 to 90 days of arrival to the United States [5]. This comprehensive examination screens for infectious and non-communicable diseases and serves as a key mechanism for connecting refugees with routine and specialty care. Surveillance data from the DME provides an overview of the prevalence of a broad range of conditions likely associated with health status before resettlement in the United States.

From 2010 to 2011, CDC recommended hepatitis C screening during the DME for those with risk factors such as injection and intranasal drug use, chronic hemodialysis, HIV infection, signs or symptoms of liver disease, household contact with someone infected with HCV, or history of female genital mutilation or cutting [6]. Starting in 2012, hepatitis C screening was also recommended for refugees born between 1945 and 1965. Additionally, the guidelines stated that it was reasonable to screen all adults (≥ 18 years of age) who originated from or had lived in countries with high-moderate (2–5%) or high (≥ 5%) hepatitis C prevalence. Hepatitis C screening is not routinely recommended for children < 18 years old unless they have risk factors. In 2020, the CDC updated its hepatitis C screening guidelines for newly arrived refugees [6]. This update included universal hepatitis C screening for all new adult arrivals (≥ 18 years of age). Hepatitis C screening is also recommended for all pregnant people during each pregnancy, unaccompanied refugee minors, children with risk factors, and children born to mothers with hepatitis C.

### Hepatitis C Prevalence and Risk Factors Among Refugees

A systematic review on the prevalence of hepatitis C antibodies among immigrants and refugees estimated an overall prevalence of 19 per 1,000 individuals (range: 14–27) [7]. Country of origin-based estimates of hepatitis C prevalence in refugees currently residing in the United States are sparse and inconsistent. Recent estimates for hepatitis C antibody prevalence among Somali refugees range from 8.5 to 91 per 1,000 [8, 9]. Chronic HCV prevalence was 5.1 per 1,000 (range: 0–18) for Hmong people in a camp in Laos compared to 72.3 per 1,000 (range: 52–93) among Hmong refugees in Thailand [8].

In regions with high endemicity, most infection results from iatrogenic exposure, such as contaminated needles, medical procedures, or receipt of unscreened contaminated blood products [7]. Previous research on refugees arriving in the United Kingdom indicates a higher odds of HCV infection among those ≥ 50 years (6.71, 2.67–16.87, p < 0.001) compared to those 15–24 years, as well as increased odds of HCV infection among those with a history of blood transfusion (5.19, 1.70–15.88, p = 0.004) [10]. To better estimate the prevalence of hepatitis C, six refugee screening sites collaborated to evaluate antibody screening results at the DME.

### Study Objectives

The three main study objectives were to (1) estimate the prevalence of hepatitis C antibodies among newly arrived refugees seen at six sites in the United States from 2010 to 2017, (2) identify demographic correlates of hepatitis C antibody positivity, including country of origin, sex, and age, and (3) develop a predictive statistical model of hepatitis C antibody positivity using these demographic correlates to estimate missed cases of hepatitis C antibody positivity among unscreened refugees in the study population. Results could be used to inform more targeted screening guidelines. Furthermore, results could be used to estimate the number of additional cases of antibody positivity that could have been identified using CDC’s updated hepatitis C screening guidelines.

## Methods

### Study Design

A retrospective cross-sectional study examined hepatitis C screening and prevalence among newly arrived refugees to the United States between January 1, 2010, and December 31, 2017.

### Setting

Six sites contributed data for the analysis, including four state refugee health programs and two clinics that perform the DME. Partners included the state refugee health programs in Colorado, Minnesota, Texas, and Washington, and refugee health clinics in Denver, Colorado and Philadelphia, Pennsylvania.

### Population

The analysis included refugees, asylees, Cuban/Haitian entrants, Amerasians, Special Immigrant Visa holders, unaccompanied refugee minors, and certified victims of human trafficking who arrived between 2010 and 2017 at one of the six participating sites (N = 144,905). In this analysis, the people arriving to the United States under the visa statuses listed previously are collectively referred to as “refugees”. Refugees screened more than one year after their U.S. arrival date were excluded from the analysis. The results submitted from the participating six sites represented approximately 27.6% of all refugees arriving to the United States from 2010 to 2017 [4].

### Variables

Demographic data included nationality, WHO region of origin (Africa, the Americas, Eastern Mediterranean, Europe, South-East Asia, and the Western Pacific), sex (male or female), and age at the DME [11]. Five sites provided data on an individual’s nationality, while one site could only provide the country of birth and the country of departure. In this case, nationality was extrapolated using an algorithm that combined ethnicity, language, country of birth, and country of departure. A categorical variable was derived for the prediction model, containing individual categories for the 14 countries with the largest number of refugees and the rest combined into one “other” category as outlined in Table 1. Refugees with the missing country of departure, country of birth, and nationality were excluded from the analysis. Data were excluded when the country of departure, country of birth, or nationality were listed as the United States. Age categories were defined as 0–4 years, 5–12 years, 13–17 years, 18–44 years, 45–64 years, and ≥ 65 years. In the prediction model, the 18-44-year age group was divided into different categories (18–23, 24–29, 30–35, and 36–44 years).

**Table 1:** Demographic characteristics among newly arrived refugees screened for hepatitis C antibody at six refugee health clinics in the United States, 2010–2017

	Screened for anti-HCV n (%)	Not Screened for anti-HCV n (%)	Total^a^ n (%)	*p*
**Nationality**				< 0.01
Afghanistan	3,612 (37.8)	5,935 (62.2)	9,547 (7.2)	
Bhutan	5,890 (65.3)	3,129 (34.7)	9,019 (6.8)	
Burma	18,078 (68.9)	8,175 (31.1)	26,253 (19.8)	
Burundi	368 (60.0)	245 (40.0)	613 (0.5)	
Cuba	4,157 (18.9)	17,833 (81.1)	21,990 (16.6)	
Democratic Republic of Congo	3,921 (54.7)	3,249 (45.3)	7,170 (5.4)	
Eritrea	1,349 (50.2)	1,336 (49.8)	2,685 (2.0)	
Ethiopia	1,525 (56.2)	1,188 (43.8)	2,713 (2.1)	
Iran	1,533 (47.9)	1,670 (52.1)	3,203 (2.4)	
Iraq	9,472 (43.9)	12,126 (56.1)	21,598 (16.3)	
Moldova	394 (66.8)	196 (33.2)	590 (0.4)	
Somalia	6,569 (49.6)	6,673 (50.4)	13,242 (10.0)	
Syria	965 (48.8)	1,012 (51.2)	1,977 (1.5)	
Ukraine	2,298 (67.8)	1,093 (32.2)	3,391 (2.6)	
All Others	4,572 (53.9)	3,926 (46.1)	8,498 (6.4)	
**Sex**				< 0.01
Male	34,552 (48.4)	36,858 (51.6)	71,410 (53.9)	
Female	30,151 (49.4)	30,928 (50.6)	61,079 (46.1)	
**Age at domestic medical exam (years)**				< 0.01
0–4	5,645 (38.0)	9,288 (62.0)	14.873 (11.2)	
5–12	8,578 (41.5)	12,101 (58.5)	20,679 (15.6)	
13–17	5,160 (49.2)	5,338 (50.8)	10,498 (7.9)	
18–44	37,044 (52.5)	33,453 (47.5)	70,497 (52.2)	
45–64	6,796 (50.8)	6,592 (49.2)	13,388 (10.1)	
65+	1,480 (57.9)	1,074 (42.1)	2,554 (1.9)	
Total	64,703 (48.8)	67,786 (51.2)	132,489 (100.0)	

^a^Only includes refugees who received a domestic medical examination (N = 132,489)

Screening for hepatitis C antibodies was defined as receiving laboratory screening for hepatitis C antibodies at the DME. Positive hepatitis C antibodies were defined as the presence of hepatitis C antibodies in laboratory evaluation performed at the DME. Indeterminate hepatitis C antibody results were excluded from prevalence and prevalence ratio analyses.

### Data Source

Demographic and screening data were obtained from the individual site’s refugee health screening databases for the four refugee state health programs or electronic medical records for the two clinics. Data including personally identifiable information, such as name and date of birth, were removed at the host sites and combined for analysis.

### Statistical Analysis

Descriptive statistics were conducted. Chi-squared tests were calculated for univariate analysis of baseline data. We performed a multiple binary logistic regression to calculate prevalence ratios and 95% confidence intervals (95% CI) to identify demographic factors associated with hepatitis C antibodies (WHO region of origin, sex, age). We evaluated the regression model for significant interactions, and the number of variables was reduced using backward elimination and likelihood ratio tests to determine the best model. Region of origin was used for the regression model due to smaller sample sizes within countries of origin. We used a multiple binary logistic regression model to estimate the number of cases of hepatitis C antibody positivity in the study sample who were not screened. Demographic variables and related risk factors were considered potential predictors, along with the screening site. The best-fit model was selected by AIC value. We utilized bootstrap validation to calculate an adjusted c-statistic.

### Ethical Review

This study using retrospective de-identified health data was determined exempt from review under category IV by the University of Minnesota Institutional Review Board (IRB) (Study number 1605E87211), the Washington State IRB (Project number E-041516-H), and the Colorado Multiple IRB (Protocol number 18–0883); Thomas Jefferson University’s IRB approved this study under an expedited review (12 F.563). A CDC human subjects advisor determined that this project did not meet the definition of human subjects research under 45 CFR 46.102(d).

## Results

### Population Description

The participating sites submitted records for a combined total of 144,905 refugee arrivals. Among these, 153 were excluded due to missing or discrepant data. Among the remaining 144,752 records, 132,489 (91.5%) received a DME. Among those who received a DME, 64,703 (48.8%) were screened for hepatitis C antibodies (Table 1). The proportion of refugees screened by participating clinics ranges varied. Among clinics that performed at least 100 DMEs: 32% of clinics tested < 25% of arrivals for hepatitis C antibodies, and 48% of clinics tested ≥ 75% of arrivals for hepatitis C antibodies. Screening for hepatitis C antibodies increased with age; 34.6% of those 0–4-years were screened at the DME compared to 54.2% of those ≥ 65 years. Among refugees born from 1945 to 1965, 51% of those who received a DME after 2012 were tested for hepatitis C antibody, which is when CDC recommended universal screening for this cohort.

### Hepatitis C Antibody Prevalence

Of the 64,703 refugees tested for hepatitis C antibodies at their DME, 1,030 (1.6%) had a positive hepatitis C antibody result (Table 2). Thirty-four (< 0.1%) had an indeterminate hepatitis C antibody result and were excluded from subsequent analyses. Among those ≥ 18 years at the time of screening, 912 (2.0%) tested positive.

**Table 2:** Prevalence of hepatitis C antibodies by nationality among newly arrived refugees at six refugee health clinics in the United States, 2010–2017

	Total			Among those ≥ 18 years		
	Screened for anti-HCV^a^ n	Positive anti-HCV n (%)	Negative anti-HCV n (%)	Screened for anti-HCV^a^ n	Positive anti-HCV n (%)	Negative anti-HCV n (%)
**Nationality**						
Afghanistan	3,612	23 (0.6)	3,589 (99.4)	2,549	19 (0.7)	2,530 (99.3)
Bhutan	5,889	30 (0.5)	5,859 (99.5)	4,558	26 (0.6)	4,532 (99.4)
Burma	18,059	511 (2.8)	17,548 (97.2)	11,706	472 (4.0)	11,234 (96.0)
Burundi	368	20 (5.4)	348 (94.5)	199	17 (8.5)	182 (91.5)
Cuba	4,156	24 (0.6)	4,132 (99.4)	3,591	22 (0.6)	3,569 (99.4)
Democratic Republic of Congo	3,917	126 (3.2)	3,791 (96.8)	2,160	103 (4.8)	2,057 (95.2)
Eritrea	1,348	14 (1.0)	1,334 (99.0)	1,055	13 (1.2)	1,042 (98.8)
Ethiopia	1,525	19 (1.2)	1,506 (98.8)	1,152	17 (1.5)	1,135 (98.5)
Iran	1,533	8 (0.5)	1,525 (99.5)	1,312	6 (0.5)	1,306 (99.5)
Iraq	9,471	49 (0.5)	9,422 (99.5)	6,812	39 (0.6)	6,773 (99.4)
Moldova	394	15 (3.8)	379 (96.2)	319	15 (4.7)	304 (95.3)
Somalia	6,563	73 (1.1)	6,490 (98.9)	4,202	62 (1.5)	4,140 (98.5)
Syria	965	3 (0.3)	962 (99.7)	530	1 (0.2)	529 (99.8)
Ukraine	2,298	46 (2.0)	2,252 (98.0)	1,846	43 (2.3)	1,803 (97.7)
All others	4,571	69 (1.5)	4,502 (98.5)	3,305	57 (1.7)	3,248 (98.3)
**Sex**						
Male	34,529	602 (1.7)	33,927 (98.3)	24,531	532 (2.2)	23,999 (97.8)
Female	30,140	428 (1.4)	29,712 (98.6)	20,765	380 (1.8)	20,385 (98.2)
**Age at domestic medical exam (years)**						
0–4	5,644	28 (0.5)	5,616 (99.5)			
5–12	8,572	49 (0.6)	8,523 (99.4)			
13–17	5,157	41 (0.8)	5,116 (99.2)			
18–44	37,033	630 (1.7)	36,403 (98.3)			
45–64	6,786	218 (3.2)	6,568 (96.8)			
65+	1,477	64 (4.3)	1,413 (95.7)			
Total	64,669	1,030 (1.6)	63,639 (98.4)	45,296	912 (2.0)	44,384 (98.0)

^a^Excludes those with an indeterminate anti-HCV result

#### Nationality

Among countries of origin with at least 10 individuals who tested positive for hepatitis C antibodies, the highest prevalence was observed among refugees from Burundi (5.4%), Moldova (3.8%), the Democratic Republic of Congo (3.2%), Burma (2.8%), and Ukraine (2.0%). Among those 18 years and older at the time of screening, the prevalence was highest among those from Burundi (8.5%), the Democratic Republic of Congo (4.8%), Moldova (4.7%), Burma (4.0%), and Ukraine (2.3%).

#### Sex

Among the 30,140 females screened for hepatitis C antibodies (excluding 11 with an indeterminate result), 428 (1.4%) tested positive. Among the 34,529 males screened for hepatitis C antibodies (excluding 23 with an indeterminate result), 602 (1.7%) tested positive. Among those 18 years and older, 380 (1.8%) females and 532 (2.2%) males tested positive.

#### Age

Among children younger than 5 years, 0.5% had hepatitis C antibodies detected, compared to 1.7% of adults 18–44 years, 3.2% of adults 45–64 years, and 4.3% of adults ≥ 65 years.

### Hepatitis C Risk Factors

Compared to refugees from the Eastern Mediterranean region, refugees from the Americas had a 41% lower prevalence of hepatitis C antibodies (95% CI, 0.38–0.90), after adjusting for age and sex (Table 3). Refugees from Africa, South-East Asia, Western Pacific, and Europe had 3.6 (95% CI 3.0-4.4), 2.9 (2.5–3.5), 2.6 (1.2–5.9), and 2.4 (1.8–3.2) times higher adjusted prevalence of hepatitis C antibodies compared to refugees from the Eastern Mediterranean, respectively.

**Table 3:** Factors associated with hepatitis C antibody detection among newly arrived refugees at six refugee health clinics in the United States, 2010–2017

	Screened for anti-HCV^a^	Positive anti-HCV	Negative anti-HCV	Unadjusted PR (95% CI)	Adjusted PR^b^ (95% CI)
**World Health Organization region of origin**					
Africa	8,247	207 (2.5)	8,040 (97.5)	3.4 (2.8–4.1)	3.6 (3.0-4.4)
Americas	4,544	24 (0.5)	4,520 (99.5)	0.7 (0.5–1.1)	0.6 (0.4–0.9)
Eastern Mediterranean	23,474	175 (0.7)	23,299 (99.3)	Reference	Reference
Europe	3,295	71 (2.2)	3,224 (97.8)	2.9 (2.2–3.8)	2.4 (1.8–3.2)
South-East Asia	24,873	547 (2.2)	24,326 (97.8)	2.9 (2.5–3.5)	2.9 (2.5–3.5)
Western Pacific	236	6 (2.5)	230 (97.5)	3.2 (1.5–7.6)	2.6 (1.2–5.9)
**Sex**					
Male	34,529	602	33,927	1.2 (1.1–1.4)	1.2 (1.1–1.4)
Female	30,140	428	29,712	Reference	Reference
**Age at domestic medical exam (years)**					
0–4	5,644	28 (0.5)	5,616 (99.5)	Reference	Reference
5–12	8,572	49 (0.6)	8,523 (99.4)	1.2 (0.7–1.8)	1.2 (0.7–1.9)
13–17	5,157	41 (0.8)	5,116 (99.2)	1.6 (1.0-2.6)	1.58 (1.0-2.55)
18–44	37,033	630 (1.7)	36,403 (98.3)	3.4 (2.35-5.0)	3.6 (2.5–5.3)
45–64	6,786	218 (3.2)	6,568 (96.8)	6.5 (4.4–9.6)	7.1 (4.8–10.5)
65+	1,477	64 (4.3)	1,413 (95.7)	8.7 (5.6–13.6)	9.1 (5.8–14.1)

^a^Excludes those with an indeterminate anti-HCV result

^b^Adjusted for age at domestic medical exam, sex, and WHO region of origin

After adjustment for age and region of origin, males had a 25% increased prevalence of hepatitis C antibodies (95% CI 1.1–1.4) compared to females. Compared with children aged ≤ 5 years, refugees 18–44 (3.6 (95% CI 2.5–5.3)), 45–64 (7.1 (95% CI 4.8–10.5)), and ≥ 65 (9.1 (95% CI 5.8–14.1)) had a higher adjusted prevalence of hepatitis C antibodies. The adjusted prevalence of hepatitis C antibodies was not significantly higher for children 5–12 years or 13–17 years compared to children aged 5 years and younger.

### Predictive Statistical Model

The sample was restricted to refugees ≥ 18 years. The selected model included age (Χ2 = 466.5, p < 0.001), sex (Χ2 = 196.5, p < 0.001), nationality (Χ2 = 9.7, p = 0.002), and the collaborating site (Χ2 = 29.7, p < 0.001) (adj. c-statistic = 0.75). Among adult refugees in this population who were not screened for hepatitis C antibodies during the DME, an estimated 1.2%, or 498 people, would have tested positive.

## Discussion

### Hepatitis C Screening

During 2010–2017, CDC DME guidelines only recommended hepatitis C screening for those with risk factors such as injection drug use or HIV infection and, starting in 2012, for those born from 1945 to 1965 [6]. The guidelines stated that it was reasonable to screen adults from countries with a hepatitis C prevalence ≥ 2%. Due to these recommendations, only 48.8% of refugees screened at the participating sites were tested for hepatitis C antibodies at their DME. At least 65.2% of those who received a DME would have been eligible for hepatitis C antibody screening under the 2020 CDC guidelines. An estimated 498 persons with hepatitis C antibody positivity were missed among unscreened adults. As a comparison, CDC recommends screening all refugees aged 13 to 64 years for HIV. Previous research indicates that 85.2% of adult refugees were screened for HIV among nine sites conducting the DME between 2014 and 2016 [12]. This emphasizes both the value added to screen all adult refugees for hepatitis C at the DME and the potential to increase screening based on other screening recommendations.

The proportion of those screened for hepatitis C antibodies during their DME varied significantly based on country of origin and age. Adults and those from higher incidence countries were screened at higher proportions. Males were also screened at a significantly higher proportion than females, which may have been due to risk-factor-based screening. Screening for hepatitis C is important for secondary prevention to reduce the long-term risk of cirrhosis and HCC [13].

### Hepatitis C Antibody Prevalence

Adult refugees ≥ 18 years, in addition to males, had a significantly higher prevalence of hepatitis C antibodies. Refugees from Africa, Southeast Asia, Western Pacific, and Europe had a significantly higher prevalence of HCV antibodies than refugees from the Eastern Mediterranean. In contrast, refugees from the Americas had a significantly lower prevalence of hepatitis C antibodies than refugees from the Eastern Mediterranean. Adult refugees coming from Burundi, the Democratic Republic of Congo, Moldova, Burma, and Ukraine all had a crude hepatitis C antibody prevalence higher than 2%. A 2014 review of existing studies on global hepatitis C prevalence reported similar hepatitis C antibody prevalence for adults from the Democratic Republic of Congo (4.3%), Moldova (4.5%), Ukraine (3.6%), and Burma (1.7%) [14]. However, the estimated adult prevalence for Burundi was only 1-1.5%, which was lower than we observed in our study population [14]. Refugees may have a different risk of hepatitis C compared to others of the same nationality since many refugees have spent years living outside of their country of origin prior to resettlement in the United States, and they are often of different demographics and exposure risk than the general population [15, 16].

### Hepatitis C Follow-up Care

The current DME guidelines recommend an HCV RNA polymerase chain reaction (PCR) test for refugees with a positive HCV antibody test [5]. People with a positive hepatitis C antibody test and negative HCV RNA test do not have hepatitis C. Positive hepatitis C antibodies and negative HCV RNA occur in the setting of resolved infections and false positive antibody screening tests [17]. CDC guidance recommends people with positive HCV RNA receive care management by a healthcare provider with expertise in chronic liver disease and hepatitis C [5]. Other recommendations for management of persons diagnosed with hepatitis C include vaccination for hepatitis A and hepatitis B, hepatitis C direct-acting antiviral (DAA) treatment, and ongoing management. CDC guidance also recommends that health education should be provided to patients with HCV infection, including prevention of transmission, signs and symptoms of cirrhosis, and HCC. The current American Association for the Study of Liver Diseases guidelines should be followed for treatment [18]. Linkage to care is an important next step for those with chronic hepatitis C. It is important to train healthcare providers on testing procedures and asking patients about hepatitis C risk factors [19]. Compared to private health insurance, Medicaid was associated with lower odds of being treated [20, 21]. However, treatment rates improved after Medicaid removed the specialist provider and advanced fibrosis restriction requirements for treatment. Without access to treatment and follow-up care, those with hepatitis C are at higher risk for end-stage liver disease [22]. Previous research indicates the cost-effectiveness of newer DAA treatments [23, 24].

### Study Strengths and Limitations

This analysis is subject to several limitations. Refugees arriving to regions of the United States not included in this evaluation may have been underrepresented in these data. However, the refugee population included in this review represents nearly 28% of all newly arrived refugees in the United States during the study period. This study does not provide comprehensive global or regional estimations of hepatitis C prevalence in refugees because the study population was limited to countries of origin for newly arrived refugees in the United States. The prediction model is specific to this study sample and should not be applied beyond these collaborating sites. It also had only a fair c-statistic value suggesting unexplained variations in hepatitis C antibody prevalence which could affect the predicted estimate. We were unable to accurately estimate the prevalence of HCV infections, confirmed by HCV RNA, in this population due to differences in state reporting rules for HCV infections and the ability of each study site to obtain confirmatory testing results for this study. Many screening programs show a substantial decreased rate of return of patients who are identified as HCV antibody positive for confirmation of diagnosis by HCV RNA testing [25]. Reflex testing, or immediately performing an HCV RNA test on all with a positive HCV antibody using the same specimen, would improve state refugee health programs’ abilities to identify HCV infections. An improved linkage system between state refugee health databases and infectious disease surveillance systems would also allow us to better evaluate the prevalence of HCV infections confirmed by HCV RNA. Such analyses could further inform hepatitis C screening recommendations for refugees arriving in the United States by determining what percentage of refugee patients with positive hepatitis C antibodies ultimately test positive for HCV RNA and require linkage to care and treatment.

Refugee screening clinics employed differing screening protocols, which could affect prevalence estimates. However, there was no significant difference in prevalence between clinics that did and did not universally screen for hepatitis C antibodies. Additionally, it was not until 2012 that CDC recommended that all adults in the U.S. born from 1945 to 1965 receive universal screening for hepatitis C antibodies, which was midway through this study period. Even after this recommendation was in place, only 51% of refugees in this birth cohort were tested for hepatitis C antibodies at the DME. There was variation in the hepatitis C risk factor data that sites collected and included in this analysis. Future work should prioritize collecting standardized risk factor data based on CDC’s DME screening guidance including HIV in addition to longitudinal follow-up data beyond the DME. The updated CDC hepatitis C screening guidelines for refugees may ensure a more standard approach to hepatitis C screening in these populations. Finally, we could not determine the method of exposure to hepatitis C, which could be valuable for prevention.

## Conclusions

The DME represents an opportunity to screen refugees for hepatitis C antibodies and complete a diagnostic HCV RNA test. DME updates in 2020 recommend universal hepatitis C screening for all new adult arrivals, all pregnant people during each pregnancy, all unaccompanied refugee minors, children with risk factors, and children born to mothers with hepatitis C. The findings demonstrate the highest prevalence of hepatitis C antibodies among adult refugees from Burundi, Moldova, Democratic Republic of Congo, Burma, and Ukraine. Future evaluations are needed to identify the prevalence of infection based on diagnostic testing and the rate of linkage to care and treatment among HCV-infected refugees.
